# Prevalence and imaging characteristics of detectable tonsilloliths on 482 pairs of consecutive CT and panoramic radiographs

**DOI:** 10.1186/1472-6831-13-54

**Published:** 2013-10-14

**Authors:** Masafumi Oda, Shinji Kito, Tatsurou Tanaka, Ikuko Nishida, Shuji Awano, Yuko Fujita, Katsura Saeki, Shinobu Matsumoto-Takeda, Nao Wakasugi-Sato, Manabu Habu, Shinya Kokuryo, Masaaki Kodama, Takeshi Kaneuji, Daigo Yoshiga, Ikuya Miyamoto, Shun Nishimura, Yoshihiro Yamashita, Kenshi Maki, Kazuhiro Tominaga, Izumi Yoshioka, Toshihiro Ansai, Yasuhiro Morimoto

**Affiliations:** 1 Division of Oral and Maxillofacial Radiology, Kyushu Dental University, Kitakyushu, Japan; 2 Division of Developmental Stomatognathic Function Science, Kyushu Dental University, Kitakyushu, Japan; 3 Division of Community Oral Health Development, Kyushu Dental University, Kitakyushu, Japan; 4 Division of Maxillofacial Surgery, Kyushu Dental University, Kitakyushu, Japan; 5 Division of Oral Medicine, Kyushu Dental University, Kitakyushu, Japan; 6 Department of Oral and Maxillofacial Surgery, Miyazaki University, Miyazaki, Japan; 7 Center for Oral Biological Research, Kyushu Dental University, Kitakyushu, Japan

**Keywords:** CT, Panoramic radiographs, Prevalence, Tonsilloliths

## Abstract

**Background:**

Recent studies suggest that tonsilloliths are clinically related to halitosis and tonsillar abscess. Based on our empirical knowledge, tonsilloliths are relatively commonly encountered in daily clinical practice. It has been reported that the detection rate of tonsilloliths was under 24% in previous reports, although experience suggests otherwise. The purpose of the study was to evaluate the prevalence and characteristics of tonsilloliths using computed tomography (CT). In addition, the possible causes of low detection rates on panoramic radiographs were evaluated based on comparisons between CT images and panoramic radiographs in order to elucidate the limitations of visualizing the area around the palatine tonsils on panoramic radiographs.

**Methods:**

482 pairs of CT images and panoramic radiographs were retrospectively assessed with respect to the presence and characteristics of tonsilloliths. In addition, the causes in cases of disagreement between the two modalities were analyzed.

**Results:**

The detection rate of tonsilloliths was 46.1% using CT scans, unlike previous reports. The characteristics of tonsillolith were dot-like figures with about 300-500 Hounsfield units within the palatine tonsil under the soft palate. The most common length of tonsilloliths was about 3 or 4 mm. As the subjects aged, the detection rate increased gradually. A significant difference in the tonsillolith detection rate was found between the over and under 40-year-old groups (p < 0.0001). However, the detection rate of tonsilloliths was only 7.3% on panoramic radiographs. A significant correlation was observed between the detection rate of tonsilloliths on panoramic radiographs and CT number (Spearman r = 0.429), size, (Spearman r = 0.318), and number of tonsilloliths (Spearman r = 0.333).

**Conclusion:**

The present results suggest that tonsilloliths are relatively more common than previously suggested. However, panoramic radiographs detect only a small percentage of palatine tonsilloliths. The low detection rates on panoramic radiographs might be related to the degree of calcification, size, and number of tonsilloliths.

## Background

Tonsilloliths are oropharyngeal concretions stemming from a reactive foreign nidus such as bacteria and organic debris within a palatine tonsillar crypt. Based on empirical knowledge, tonsilloliths are reported to be relatively commonly encountered in daily clinical practice, but patients rarely have complaints related to them. It has been reported that the detection rate of tonsilloliths was under 25% [1-4], although experience suggests otherwise. Furthermore, recent studies suggest that tonsilloliths are clinically related to halitosis and tonsillar abscess [5-7]. Therefore, the detection rate, number, size, location, calcification level, and other characteristics of tonsilloliths should be precisely understood. However, CT was not used and/or the sample size was relatively small in the previous reports [1-4].

In the present study, the detection rate, number, size, location, and calcification level of tonsilloliths were analyzed by CT images. In addition, the possible causes of the low detection rates on panoramic radiographs were elucidated based on comparing the CT images and panoramic radiographs to elucidate the limitations of visualizing the area around the palatine tonsils on panoramic radiographs.

## Methods

This study was based on 482 pairs of panoramic radiographs and CT scans that were obtained at the Division of Oral and Maxillofacial Radiology in Kyushu Dental University Hospital between 2010 and 2011 from patients with oral and maxillofacial region diseases (201 males, 281 females; mean age, 52.9 years; range, 5 to 94 years). Approval of the present study was obtained from the institutional review board of Kyushu Dental University (No. 12-19).

Panoramic radiographs were acquired using a panoramic AUTO-1000 EX system (Asahi Roentgen Ind. Co., Ltd., Kyoto, Japan). Images were taken in the incisive occlusion position holding the head by head supports with the FH plane parallel to the ground. CT was performed with an Activion 16 (Toshiba Co. Ltd., Tokyo, Japan). CT images were taken in the axial plane with 3-mm-thick contiguous sections from under the level of the orbit to under the hyoid bone to evaluate approximately the same regions viewed on panoramic radiographs. Images were obtained with standard algorithms and soft-tissue windows.

The presence of tonsilloliths was evaluated both on panoramic radiographs and CT images independently. Using the differential diagnosis of tonsilloliths on panoramic radiographs reported by Ram et al, a radiopaque nodular mass or masses piled up on the mandibular ramus and soft palate were defined as tonsilloliths [8]. The 482 pairs of images were assessed by a single, experienced dental radiologist (M. O.). The radiologist then assessed the CT and the panoramic radiographs of the same patients separately, without knowing the relationship between the two.

At the same time, the numbers, shapes, size, locations, and calcification levels of the tonsilloliths were also evaluated on both panoramic radiographs and CT scan. The masses or high density structures within the palatine tonsil were counted on the images. On CT scan, the tonsilloliths were diagnosed based on structures with over 100 Hounsfield Units (HU). The long axes of the radiopaque masses or high-density structures were measured. If the subject had multiple tonsilloliths, the size, shapes, location, and calcification levels of the largest calculus were analyzed. The locations of the tonsilloliths were divided into 3 groups (under the soft palate; coincident with the soft palate; over the soft palate) on the right or left, respectively. At the same time, the locations were divided into 2 groups, the medial and lateral halves of the lateral palatine tonsil determined based on the midline of the lateral palatine tonsil. The calcification levels of the tonsilloliths were evaluated as the maximum HU value in the density changing area.

Next, in cases of disagreement between the CT images and panoramic radiographic findings, the causes for disagreement were analyzed based on the CT findings as the gold standard. The difference in the numbers, shapes, size, locations, and calcification levels of the tonsilloliths were examined using statistical analyses of the cases in which there were and was not agreement.

All statistical analyses were performed using SPSS version 11 statistical software (SPSS, Chicago, Illinois, USA). Categorical variables were compared by the χ^2^ test. The relationships between categorical variables were assessed using Pearson’s correlation coefficient or Spearman’s rank correlation. Results were considered significant if p < 0.05.

## Results

### Detection of tonsilloliths by CT images

**Figure 1:**
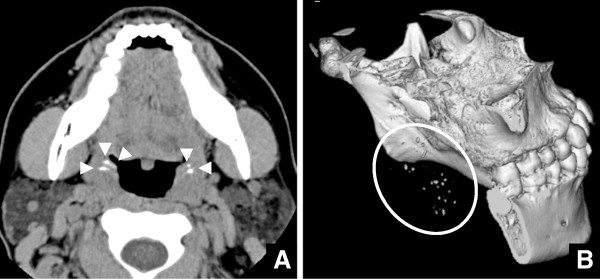
**CT images of a 31-year-old female with 28 tonsilloliths. A)** Axial CT images at the palatine tonsillar level. Twenty-eight tonsilloliths (arrowheads) are seen as high-density, dot-like structures. **B)** Three-dimensional reconstruction images of CT. Twenty-eight tonsilloliths within a circle are shown as high density dot-like structures.

**Figure 2:**
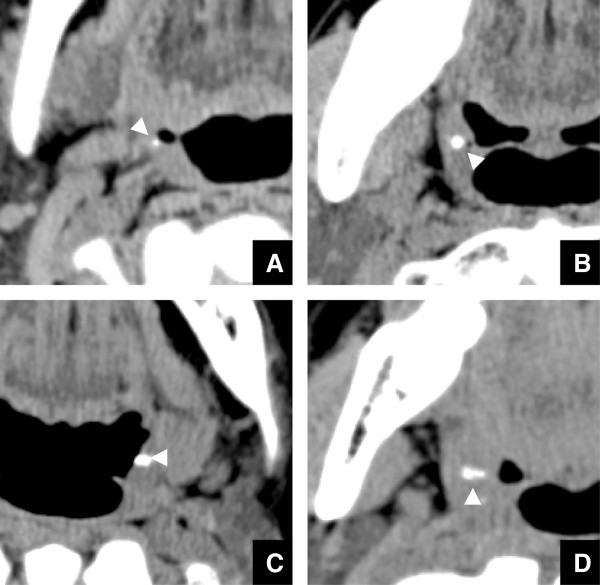
**Four kinds of tonsillolith shapes on CT images. A)** A dot shape of tonsilloliths (arrowhead) is shown on axial CT images. **B)** A round shape of tonsilloliths (arrowhead) is shown on axial CT images. **C)** An oval shape of tonsilloliths (arrowhead) is shown on axial CT images. **D)** An irregular shape of tonsilloliths (arrowhead) is shown on axial CT images.

Of the 482 individuals, 222 (46.1%) were judged to have tonsilloliths on CT scans (Table 1). The 222 patients with tonsilloliths on CT images consisted of 91 males and 131 females, and no difference by sex in the tonsillolith detection rate was seen. However, a significant correlation was found between age and the tonsillolith detection rate (Spearman r = 0.176, p < 0.0001). As the subjects aged, the detection rate increased gradually. In particular, a significant difference in the tonsillolith detection rate was found between the over and under 40-year-old groups (p < 0.0001), but the tonsillolith detection rate was relatively stable among the various groups (Table 2). Overall, 99 patients had one calculus, 37 had two, 27 had three, 21 had four, 12 had five, and so on (Table 3), up to a maximum of 28 (Figure 1). The shapes of tonsilloliths varied, including dot, round, oval, and irregular structures (Figure 2). Round or oval tonsilloliths with a long diameter under 2 mm were considered dots, while those with a long: short diameter ratio of under 3:2 were considered round, those with a long: short diameter ratio of over 3:2 were considered oval, and those with shapes that were neither round nor oval were considered irregular. The most common shape was the dot lump and/or lumps of structure(s) with high CT number(s) within the palatine tonsil on CT images. With respect to the sizes of the tonsilloliths, 59 were under 2 mm, 149 were over 2 mm and under 5 mm**,** 14 were over 5 mm and under 10 mm, and none were over 8 mm (Table 4). The most common length was from under 3 to 4 mm on CT images. On CT, 63 (13.1%) were on the right side, 78 (16.3%) were on the left side, and 81 (16.8%) were bilateral (Table 5); the rates were almost equal, with no significant difference observed (p = 0.194). Furthermore, 157 were under the soft palate, 95 were coincident with the soft palate, and 0 were over the soft palate. At the same time, 95 were in the medial half and the others were in the lateral half of the tonsil. The calcification levels of tonsilloliths on CT images were over 100 HU and under 300 HU in 95, over 300 HU and under 500 HU in 68, over 500 HU and under 1000 HU in 46, and over 1000 HU in 13 (Table 6).

**Table 1:** Sex differences in the detection of tonsilloliths on CT and panoramic radiographs

**Sex**	**CT**		**Panoramic radiographs**	
	**Presence**	**Absence**	**Presence**	**Absence**
Male	91 (18.9%)	110 (22.8%)	14 (2.9%)	187 (38.8%)
Female	131 (27.2%)	150 (31.1%)	23 (4.8%)	258 (53.5%)
Total	222 (46.1%)	260 (54.0%)	37 (7.7%)	445 (92.3%)
CT: Computed tomography				(n = 482)

**Table 2:** Age distribution in the detection of tonsilloliths on CT

**Ages**	**Number of patients**		**Detection rate (%)**
	**Presence**	**Absence**	
<9	2	2	50.0
10-19	4	22	15.5-4
20-29	20	40	33.3
30-39	20	39	33.9
40-49	28	25	52.8
50-59	32	29	52.5
60-69	40	39	50.6
70-79	51	45	53.7
80-89	23	17	57.5
>90	2	3	40.0
Total	222	260	46.1
CT: Computed tomography			(n = 482)

**Table 3:** Distribution of number of tosilloliths on CT

**Number of tonsilloliths**	**Number of patients**
0	260
1	99
2	37
3	27
4	21
5	12
6	5
7	3
8	6
9	3
10	4
>11	5
CT: Computed tomography	(n = 482)

**Table 4:** Relationship between tonsilloliths detected on panoramic radiographs and their sizes on CT

**Sizes (mm) on CT**	**Number of patients detected by CT**	**Number of patients detected by PR**	**Detection rate by PR (%)**
<1	8	0	0
1-2	51	0	0
2-3	53	5	9.4
3-4	55	14	25.5
4-5	41	10	24.4
5-6	10	4	40.0
6-7	2	1	50.0
7-8	2	1	50.0
Total	222	35	15.8

CT: Computed tomography.

PR: Panoramic radiographs.

**Table 5:** Distribution of the sides of tonsilloliths detected on CT panoramic radiographs

**Sides**	**Number of patients**	
	**CT**	**Panoramic radiographs**
Right	63	14
Left	78	18
Bilateral	81	3
Total	222	35

CT: Computed tomography.

**Table 6:** Relationship between tonsilllotliths detected on panoramic rediographs and their calsification level on CT

**Calsifide level on CT (HU)**	**Number of patients detected by CT**	**Number of patients detected by PR**	**Detection rate by PR (%)**
<200	51	0	0
200-300	44	0	0
300-400	34	5	14.7
400-500	34	7	20.1
500-600	19	4	21.1
600-700	10	4	40.0
700-800	8	3	37.5
800-900	6	3	50
900-1000	3	1	33.3
100-1100	3	1	33.3
1100-1200	3	2	66.7
>1200	7	5	71.4
Total	222	35	15.8

CT: Computed tomography.

PR: Panoramic radiographs.

### Detectable tonsilloliths on panoramic radiographs

**Figure 3:**
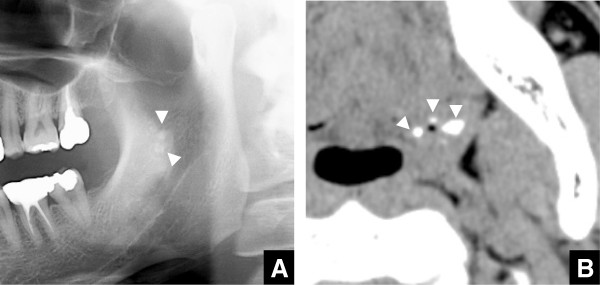
**Panoramic radiographs and axial CT images at the palatine tonsillary level of a 38-year-old male. A)** Radiopaque nodular masses are shown in the mandibular ramus on panoramic radiographs. **B)** Tonsilloliths (arrowheads) are shown as high-density structures on axial CT images.

**Figure 4:**
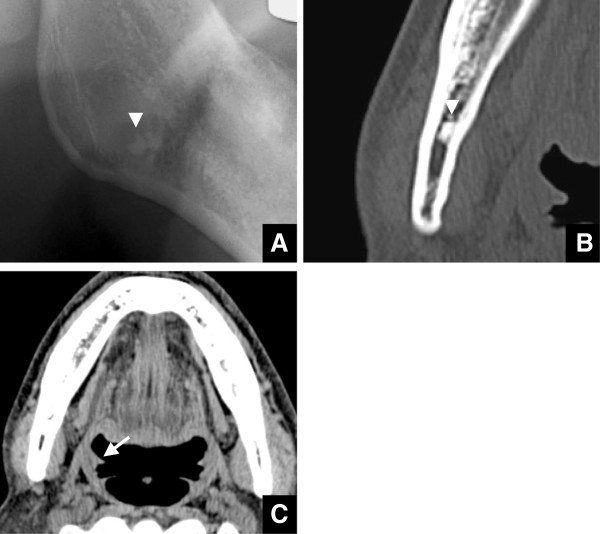
**Panoramic radiographs and axial CT images at the palatine tonsillary level of a 78-year-old male. A)** A radiopaque nodular mass is shown in the mandibular ramus on panoramic radiographs. **B, C)** An enostosis in the mandibular ramus (arrowhead), with no tonsilloliths (arrows), is shown as high-density, dot-like structures.

Of the 482 individuals, 37 (7.7%) (Figure 3) were judged to have tonsilloliths on panoramic radiographs, but 222 (46.1%) were judged to have tonsilloliths on CT scans (Table 1). A significant difference in the tonsillolith detection rate was found between the two modalities (p < 0.0001). Of the 37 patients judged to have tonsilloliths on panoramic radiographs, 2 did not actually have calcifications on CT images (Figure 4). In both cases, enostosis in the mandibular ramus was detected on CT images (Figure 4). Therefore, there were actually 35 (7.3%) patients with tonsilloliths on panoramic radiographs. Almost all tonsilloliths showed a radiopaque nodular mass or masses on panoramic radiographs. The size on panoramic radiographs was over 2 mm and under 5 mm in 29, over 5 mm and under 10 mm in 6, and over 10 mm in 0 (Table 4). Fourteen were located on the right side, 18 were on the left side, and 3 were bilateral (Table 5). At the same time, 25 were piled up on the mandibular ramus and under the soft palate, 0 were on the mandibular ramus and over the palate, and 10 were on the mandibular ramus and coincident with the soft palate on panoramic radiographs.

### Differences in the detection of tonsilloliths between panoramic radiographs and CT images

**Figure 5:**
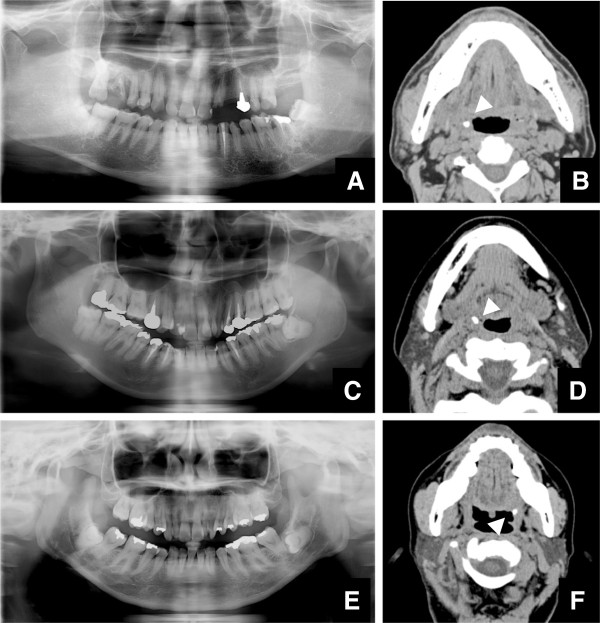
**Cases with tonsilloliths are detected on CT images, but not on panoramic radiographs. A)** Panoramic radiographs at the palatine tonsillary level of a 65-year-old male. A radiopaque nodular mass is not seen. **B)** Axial CT images at the palatine tonsillary level of the same patient as in Figure 5A. Tonsilloliths (arrowheads) are seen at the level of the mandibular teeth as high-density, oval-like structures with a long axis of 5.03 mm and 1128 HU. **C)** Panoramic radiographs at the palatine tonsillary level of a 32-year-old female. A radiopaque nodular mass is not seen. **D)** Axial CT images at the palatine tonsillary level of the same patient as in Figure 5C. Tonsilloliths (arrowheads) are seen at the level of the mandibular teeth as high-density, oval-like structures with a long axis of 5.96 mm and 912 HU. **E)** Panoramic radiographs at the palatine tonsillary level of a 48-year-old female. A radiopaque nodular mass is not seen. **F)** Axial CT images at the palatine tonsillary level of the same patient as in Figure 5E. Tonsilloliths (arrowheads) are seen at the level of the mandibular teeth as high-density, oval-like structures with a long axis of 5.35 mm and 905 HU.

There were 185 cases of disagreement between panoramic radiographic and CT imaging findings (Table 1). It was presumed that CT numbers and tonsillolith size might explain why the tonsilloliths were detected on CT images, but not on panoramic radiographs. Certainly, there was a significant correlation between CT number and the tonsillolith detection rate on panoramic radiographs (Spearman r = 0.429, p < 0.0001). The higher the CT number was, the higher the tonsillolith detection rate was. All 95 cases with CT number < 300 HU were not detected on panoramic radiographs (Table 6). Furthermore, a significant correlation was observed between size and tonsillolith detection rate on panoramic radiographs (Spearman r = 0.318, p < 0.0001). The tonsillolith detection rate was higher as size increased. In fact, in 179 of 187 cases that tonsilloliths were not detected on panoramic radiographs, calculus sizes were <5 mm (Table 4). In addition, a significant correlation was found between the tonsillolith detection rate on panoramic radiographs and the number of tonsilloliths (Spearman r = 0.333, p < 0.0001). However, in three cases, the two main causes mentioned above did not appear responsible (Figure 5). In all cases, the tonsilloliths were at a relatively low level that coincided with the mandibular ramus and the head of the submandibular glands. These cases shared no other similar characteristics (Table 7).

**Table 7:** Data summary of the three tonsilloliths that could not be detected on panoramic radiographs despite high calcification levels and relatively large sizes on CT

	**Case 1**	**Case 2**	**Case 3**
Sex	Male	Female	Female
Age (years)	65	32	48
Side	Bilateral	Right	Left
Number of tonsilloliths	3	1	2
Size (mm)	5.03	5.96	5.35
Location	Under soft palate	Under soft palate	Under soft palate
	Internal	Internal	Internal
Calcified level (HU)	1128	912	905

CT: Computed tomography.

## Discussion

One of the important results of the present study was that relatively many subjects with no symptoms in the oropharyngeal area had one or more tonsilloliths (46.1%). Some investigators have also previously reported that the detection rate of tonsilloliths was very low [1,2]. However, the data from these reports did not coincide with our clinical empirical knowledge based on CT images. This may be explained by the fact that CT images were not used in most of the previous reports on tonsilloliths [1,2]. To the best of our knowledge, the detection rate of tonsilloliths was relatively high (about 15% or 25%) in only two reports using CT images [3,4]. The sample size was relatively low (100 or 150 subjects), so that precise analysis could probably not have been done. In addition, the tonsillolith detection rate in the present study was significantly higher in subjects over 40 years old. However, the detection rates within the under 40-year-old subjects and the over 40-year-old subjects were relatively stable. In the previous reports, the conclusions on the relationship between the tonsillolith detection rate and age were contradictory. One concluded that the tonsillolith detection rate was related to age [9], while the other did not [10]. The present precise results can explain the contradictory opinions suggested by the previous reports; a very significant difference was observed in the relationship between the detection rate and age in the over and under 40-year-old groups. On the other hand, the correlation coefficient between the two may be relatively low, because the detection rates within the under 40-year-old subjects and within the over 40-year-old subjects were relatively stable.

Another of the important results is that the tonsilloliths detected on panoramic radiographs represent only a small part of the tonsillar calculi that were actually present. The tonsillolith detection rate on panoramic radiographs was only 7.3%, about one-sixth of that on CT images. The present results indicate that the main causes for the discrepancy were the calcification levels and the sizes of the tonsilloliths. In fact, calculus sizes were <5 mm in 179 of the 187 cases of tonsilloliths undetected on panoramic radiographs. In addition, almost all cases with CT number < 300 HU were not detected on panoramic radiographs. At the same time, the tonsillolith detection rate on panoramic radiographs was also significantly related to the number of tonsilloliths.

However, the causes for the discrepancy between panoramic radiographs and CT could not be elucidated in 3 cases. In 3 cases, the tonsilloliths were at a relatively low level that coincided with the mandibular roots and the heads of the submandibular glands. The tonsilloliths may have been obscured by the teeth and trabecular bones.

The location of tonsilloliths was not significantly related to the tonsillolith detection rate on panoramic radiographs. However, enostosis in the mandibular ramus was misdiagnosed as tonsilloliths on panoramic radiographs in two cases of disagreement between CT and panoramic radiographs. On panoramic radiographs, the palatine tonsil overlaps the mandibular ramus. It might be very difficult to differentiate between tonsilloliths and calcification diseases involving the mandible, such as enostosis. Therefore, based on the present results, panoramic radiographs cannot be considered to be a useful screening tool for tonsilloliths in the general dental population. In particular, CT examinations might be used for patients with clinical findings such as halitosis and a swollen palatine tonsil of unknown origin. At the same time, the characteristics of tonsilloliths on panoramic radiographs were accurately observed. The characteristic findings on panoramic radiographs of tonsilloliths were radiopaque mass(es) of various size(s) (mainly from 5 mm to 10 mm) near the soft palate, overlapping the mandibular ramus. In addition, no tonsilloliths were present over the soft palate. Less skillful dental radiologists might have more false-positive and false-negative cases of tonsilloliths on panoramic radiographs. Therefore, the particular radiological findings of tonsilloliths described above on panoramic radiographs should be highlighted in lectures for dental students and postgraduate dentists worldwide.

In the previous cases in children, only large tonsilloliths and clinical manifestations such as tonsillar abscess were described. However, even in children under 12 years old, there were some subjects (2.1%) with small tonsilloliths in the present study; thus, some tonsilloliths do occur in children. Since the presence of tonsilloliths was related to the occurrence of palatine abscess, pediatric dentists should be aware of the above.

As mentioned above, it was found that the prevalence of tonsilloliths was much greater than in previous reports [1-4]. The presence of tonsilloliths may be related to halitosis based on some previous reports [5,7]. Therefore, some unknown causes of halitosis might be related to the presence of tonsilloliths. At the same time, we are planning to elucidate the relationship between the presence of tonsilloliths and halitosis in our next study. It is important to evaluate whether tonsilloliths are present on CT examination in patients with unknown causes of halitosis. If tonsilloliths are found incidentally on CT and/or panoramic radiographs in patients without a palatine abscess or halitosis, they should be followed regularly. However, if tonsilloliths are suspected as the causes of the palatine abscess or chronic halitosis, they should be removed [3].

The limitation of this study was that the sample size was small, and the data of subjects with diseases involving the oral and maxillofacial region were limited, except for the oropharyngeal area. Thus, the present results should be interpreted as being relevant to relatively healthy, active populations. A further limitation of this study was that only Japanese subjects were examined.

## Conclusions

The present results suggest that the tonsillolith detection rate is relatively higher than suggested in previous reports. However, panoramic radiographs detected only a small percentage of palatine tonsilloliths. The differences mentioned above should be recognized. The low detection rates on panoramic radiographs might be related to the degree of calcification, the size, and the number of tonsilloliths.

## Competing interests

The authors declare that they have no competing interests.

## Authors’ contributions

MO, SK, TT, SM, NW, SN, and YM: Conceptualized and designed the study, participated in the performance of the research, participated in data analysis, drafted the initial manuscript. IN, SA, YF, KS, MH, SK, MK, TK, DY, YM, and IM: Participated in the performance of the research and data collection. KM, KT, IY, TA: Drafted the initial manuscript, critically reviewed the manuscript, and approved the final manuscript as submitted. All authors read and approved the final manuscript.

## Pre-publication history

The pre-publication history for this paper can be accessed here:

http://www.biomedcentral.com/1472-6831/13/54/prepub
